# Recent Developments and Implementations of Conductive Polymer-Based Flexible Devices in Sensing Applications

**DOI:** 10.3390/polym14183730

**Published:** 2022-09-07

**Authors:** Vinh Van Tran, Sanghyuck Lee, Daeho Lee, Thanh-Hai Le

**Affiliations:** 1 Laser and Thermal Engineering Laboratory, Department of Mechanical Engineering, Gachon University, Seongnam 13120, Korea; 2 Department of Polymer Engineering, Graduate School, Chonnam National University, Gwangju 61186, Korea; 3 Alan G. MacDiarmid Energy Research Institute, Chonnam National University, Gwangju 61186, Korea

**Keywords:** conductive polymers, flexible sensors, printing techniques, solution processing

## Abstract

Flexible sensing devices have attracted significant attention for various applications, such as medical devices, environmental monitoring, and healthcare. Numerous materials have been used to fabricate flexible sensing devices and improve their sensing performance in terms of their electrical and mechanical properties. Among the studied materials, conductive polymers are promising candidates for next-generation flexible, stretchable, and wearable electronic devices because of their outstanding characteristics, such as flexibility, light weight, and non-toxicity. Understanding the interesting properties of conductive polymers and the solution-based deposition processes and patterning technologies used for conductive polymer device fabrication is necessary to develop appropriate and highly effective flexible sensors. The present review provides scientific evidence for promising strategies for fabricating conductive polymer-based flexible sensors. Specifically, the outstanding nature of the structures, conductivity, and synthesis methods of some of the main conductive polymers are discussed. Furthermore, conventional and innovative technologies for preparing conductive polymer thin films in flexible sensors are identified and evaluated, as are the potential applications of these sensors in environmental and human health monitoring.

## 1. Introduction

Flexible and wearable sensors have attracted significant attention for monitoring environmental conditions, human physical activity, health status, and diseases because of their high flexibility, great conformability, and low cost [1]. Emerging research in nanotechnology and materials science has recently yielded numerous materials for designing and developing flexible electronic sensors. Both inorganic and organic nanomaterials, such as metal oxides, metal nanoparticles/nanowires, carbon materials, and conductive polymers (CPs), have shown potential for integration into flexible electronic devices [2]. While traditional electronic materials, such as metals and metal oxides, have poor mechanical flexibility and very limited elasticity, CPs are considered the most promising materials for fabricating flexible sensors because of their outstanding features, such as availability, flexibility, low weight, and non-toxicity [3]. In addition, CPs are easily processed by simple solution-based methods such as spin coating, drop coating, shear coating, and dip coating, as well as advanced patterning techniques such as inkjet printing, screen printing, and three-dimensional printing. A CP film with high conductivity and mechanical flexibility can be used as flexible and stretchable electrodes or active materials in flexible and wearable devices for healthcare monitoring, temperature sensing, gas sensing, and food processing.

The most popular CPs used in flexible sensors are conjugated π-CPs, which contain delocalized π-electrons that can move freely within their unsaturated backbones to produce electrical pathways for mobile charge carriers [4]. Polyacetylene (PA), polythiophene (PT), poly [3,4-(ethylenedioxy)thiophene] (PEDOT), poly(o-phenylenediamine) (PoPDA), polypyrrole (PPy), and polyaniline (PANI) are well-known CPs that are commonly used in the construction and design of flexible electronic devices because they offer biocompatibility [5], favorable electrical and mechanical properties [6,7], and reliable electrochemical stability [8,9]. However, their patterning and deposition face limitations and challenges owing to their low solubility and poor meltability [10]. Many techniques have been employed to fabricate flexible CP thin films, but these often involve multistep processes, expensive equipment, low resolution, and risks to health and the environment [6,7,11,12]. Therefore, developing advanced techniques for the large-scale production of flexible electronic devices is important [13].

Understanding the unique features of flexible materials and their deposition technologies is critical for designing and fabricating flexible sensors [14]. This study provides a comprehensive overview of common CP materials, conventional and advanced patterning CP techniques, and promising applications of flexible CP sensors. The main aim of this study is to provide a critical update on the effective strategies that have been employed in the development of CP-based flexible sensors.

## 2. CPs and Their Properties for Flexible Devices

As mentioned previously, conjugated π-CPs have often been selected for fabricating flexible sensors based on their properties. Table 1 summarizes the structures, synthesis methods, properties, and applications of several common π-CPs.

### 2.1. Polyacetylene (PA)

PA, a linear polyene chain [–(HC=CH)_n_–], is an archetypal conjugated polymer exhibiting multifaceted properties and possessing many interesting features for developing electronic devices. These include good electrical conductivity, photoconductivity, gas permeability, supramolecular assembly formation, chiral recognition, helical graphitic nanofiber formation, and liquid crystallization [15,16]. PA has the simplest structure of organic polymers that can exhibit metal-like conductivity [17]. Owing to the repeated units of two hydrogen atoms in the chemical structure of PA, the chain can be easily decorated with pendant groups by replacing these hydrogen atoms with foreign molecules to form monosubstituted or disubstituted PAs [15]. The electrical conductivity of non-doped PAs depends strongly on their conformation, with values of 10^−9^ S·cm^−1^ and 10^−6^ S·cm^−1^ for cis- and trans-PA, respectively [18]. In contrast, PAs can achieve an almost metallic conductive level (10^4^–10^5^ S·cm^−1^) via p- or n-doping [19,20,21]. Although PA has promising conductive ability, its high instability, even at room temperature, and the difficulties in its processing have considerably limited its practical applications [22,23]. Compared to other CPs with lower electrical conductivity but better stability and processability, PA has rarely been used in designing and developing flexible devices.

Methods including catalytic and non-catalytic polymerization and precursor-assisted synthesis are used to prepare PA [24]. Of these, catalytic polymerization techniques, Ziegler−Natta or Luttinger catalysis, are commonly used to polymerize acetylene and other monomers to produce PA and oligomers such as cyclooctatetraene and vinyl acetylene [25]. The catalysts must have high solubility in organic solvents and high selectivity, such as Zeigler–Natta catalysts, which are a combination of Ti(0-n-C_4_H_9_)_4_ and (C_2_H_5_)_3_A_1_, to produce highly crystalline PA films [26]. Furthermore, these techniques allow the monitoring and observation of the structures of the final PA products with variations in the temperature and catalyst content. PA can also be synthesized by radiation polymerization approaches, such as glow discharge, ultraviolet, and Y-radiation [25]. Compared with polymerization catalysis, radiation polymerization methods can avoid using catalysts and solvents; thus, they are very promising techniques for the future development of PA. In the design and development of flexible devices, PA is often hybridized or doped with different materials, such as dihexadecyl hydrogen phosphate [27], quaternized cellulose NPs [28], and Au NPs [29], to improve their conductivity. PAs are also known as acetylene black or PA black, depending on the preparation method, and they are usually applied in electrochemical biosensors and bioelectrodes.

### 2.2. Polyaniline (PANI)

PANI is one of the most promising conjugated CPs, owing to its excellent environmental stability, high processability, high and tunable electrical conductivity, and optical properties [30]. The conductivity of PANI is highly dependent on the dopant concentration and pH; it can show metal-like conductivity at pH < 3 [31]. PANI occurs in the three different forms of leucoemeraldine [(C_6_H_4_NH)_n_], emeraldine [([C_6_H_4_NH]_2_[C_6_H_4_N]_2_)_n_], and pernigraniline [(C_6_H_4_N)_n_], based on the idealized oxidation states during the polymerization of the aniline monomers [32]. The pernigraniline base (blue/violet) is fully oxidized PANI, while the leucoemeraldine base (white/clear) is completely reduced, and emeraldine (salt-green/base-blue) is half of the oxidized PANI. PANI is conductive and more stable in the emeraldine state at room temperature. The pernigraniline and leucoemeraldine forms have poor conductivity even with doping. PANI conductivity depends significantly on the preparation method, and it can be modulated by submerging the emeraldine base in an aqueous acidic solution [33]. However, the emeraldine base state is poorly soluble owing to the stiff polymer backbone and the hydrogen bonding interactions between adjacent chains; thus, it is difficult to process. Moreover, it exhibits instability at the melt-processing temperature, which limits its practical applications. Therefore, functionalized PANI and alternative PANI derivatives are often used to develop flexible devices [34,35]. Owing to its low cost, good environmental stability, excellent optical and electrical properties, and good anticorrosion and mechanical properties, PANI has attracted much attention in the design and development of commercial technologies, especially flexible electronic devices, in various fields such as organic electronics [36], biosensors [37], chemical sensors [38,39], corrosion devices [40], photovoltaic cells [41], solar cells [42], organic light emitting diodes [43], and electrorheological materials [44]. Furthermore, PANI-based nanocomposites have undergone tremendous development via the regulation of electrical properties by protonation or charge-transfer doping. Owing to the controllability of the electrical, magnetic, mechanical, and thermal properties of CP–inorganic nanocomposites [45], PANI-based composites are considered one of the most important nanocomposite materials.

For PANI synthesis, the chemical oxidation method is the most common and straightforward; this uses a doping acid and a mixture of an oxidizing agent and a monomer precursor, in which the color change of the obtained solution to green confirms PANI formation [46,47]. Although the process is simple, such conventional methods have shown significant problems regarding the use of strong acids and oxidants, such as ammonium peroxydisulfate [48,49,50]. Moreover, this conventional technique only yields irregularly shaped PANI products. In order to obtain PANI nanostructures with diameters of <100 nm, different functional molecules have been introduced during the chemical polymerization, including surfactants, liquid crystals, polyelectrolytes, nanowire seeds, aniline oligomers, or organic dopants [51]. Such agents may work as templates to promote the self-assembly of ordered “soft templates” for forming PANI nanostructures. Moreover, a side-by-side electrospinning technique can prepare PANI nanofibers with enhanced mechanical and electrical properties [52].

### 2.3. Poly [3,4-(ethylenedioxy)thiophene] (PEDOT)

PEDOT is one of the most popular CPs because of its high conductivity, good air stability, optical transparency, and simple processing [53]. PEDOT crystals have monoclinic lamellar structures consisting of inclined π-stacks, in which the electrons are lighter than the holes [54]. The PEDOT structural model has a pseudo-orthorhombic unit cell with four monomers and one tosylate ion per cell; the lattice parameters a, b, and c are 14.0, 6.8, and 7.8 Å, respectively [55]. Owing to its advantageous properties, PEDOT has been broadly applied in the design of various flexible devices in bioelectronics and energy conversion and sensors [56,57]. Because PEDOT is hydrophobic, hydrophilic surfactant additives (i.e., poly(styrenesulfonate) (PSS)) must be used to improve its aqueous processability as a thin film [58]. Consequently, PEDOT:PSS, a water-soluble CP, can be obtained by incorporating positively charged conductive conjugated PEDOT with negatively charged insulating PSS [59]. In particular, the water-soluble long molecular chains of PSS interact with the insoluble short chains of PEDOT by Coulombic forces to form grains, which induces good water dispersion of PEDOT. Nano-sized PEDOT:PSS grains (30–50 nm) are composed of tangles containing several PEDOT segments and a single PSS chain. To improve the electrical conductivity of PEDOT:PSS, many processing methods and doping agents have been employed to remove excess PSS and induce phase separation or morphological rearrangement [60]. In these processes, polar solvents such as dimethyl sulfoxide (DMSO), ethylene glycol, and co-solvents [61] or acids such as chloroplatinic acid, sulfonic acid, and mineral acids [62,63,64] have been used to increase the conductivity of PEDOT:PSS films. Moreover, electrospinning has recently offered the possibility of producing flexible 1D PEDOT:PSS nanofibers with high conductivity [65].

In general, pristine PEDOT:PSS films show an inherent direct-current electrical conductivity (less than 1.0 S·cm^−1^) [66,67], while the modified films can show substantial improvements in conductivity of 2–3 orders of magnitude, reaching 4000 S·cm^−1^ owing to doping. In addition, PEDOT:PSS films possess a typical work function of 4.8–5.4 eV and are thus usually implemented as a p-type contact layer with fast charge transfer and injection, favored for optoelectronic use devices. Moreover, doped PEDOT:PSS films exhibit higher flexibility and stretchability than undoped films [68,69]. Such films are robust against mechanical shear, impact, bending, folding, twisting, and large tensile strains of over 100% [70,71]. In conclusion, PEDOT:PSS films with morphological evolution and structural rearrangement show high conductivity, flexibility, and stretchability [59].

### 2.4. Polypyrrole (PPy)

PPy is a heterocyclic positively charged CP containing N atoms in its oxidized form; however, its conductivity can be completely lost due to overoxidation [72]. In addition, PPy is more electroactive in organic electrolytes and aqueous solutions [73]. PPy is particularly interesting among the various CPs because its monomer (pyrrole) is easily oxidized, water-soluble, commercially available, lightweight, low-cost, and bio-compatible [74]. It also exhibits good environmental stability, high conductivity, and good redox properties [75]. Therefore, PPy has been considered in numerous applications ranging from biochemical to electrochemical energy devices [76,77,78]. Furthermore, PPy presents greater flexibility than most other CPs [78], and it can be combined with other nanostructured materials such as graphene or other carbon materials to form nanocomposites, which can show significantly enhanced properties owing to their improved ion diffusion rates and increased contact surface areas [79,80]. These outstanding features make PPy a promising candidate to meet the requirement of portable and flexible electronic devices [81,82].

PPy may be conductive because of its structure of alternating single and double bonds, which create some delocalization of electron density in the molecule [83]. However, pristine PPy is an insulator with a large bandgap energy (3.16 eV) [84]. To enhance PPy conductivity, chemical and electrochemical doping methods have been employed [85,86]. In these doping processes, PPy oxidation can remove a π-electron from the neutral polymer chain, causing its structure to change from benzenoid (aromatic) to quinoid [84,87]. Consequently, doping can convert PPy into an ionic complex comprising cations and incorporated counterions. Chemical and electrochemical polymerization methods are commonly used for PPy synthesis [81]. Recently, other advanced polymerization techniques, such as ultrasonic irradiation, vapor-phase polymerization, electrospinning, microemulsion, mechanochemical polymerization, and photopolymerization, have attracted interest in the preparation of highly conductive PPy [88,89].

### 2.5. Polythiophene (PT)

PT is an important CP owing to its simple structure, high stability, and various optoelectronic properties. PT and its derivatives in both undoped and doped states have gained great attention in sensing device applications due to the structural modification and solution processability [90]. Moreover, it also exhibits a selective barrier effect to specific molecules and high adsorption affinity on the electrode surface. The overall electronic properties can be tuned using side-chain groups or dopants with a band gap from 3 to 1 eV [91]. However, the polymerization and deposition of PT on large insulating substrates have remained a big challenge because of the high oxidation potential. Several methods have been widely used for the preparation of polymeric thin films from thiophene monomers, including chemical oxidation in solution, electrochemical oxidation, and oxidative chemical vapor deposition [92]. Among them, electrochemical oxidation polymerization is the most common technique due to the easy control of the polymerization degree [93]. Moreover, PT nanofiber structures can be obtained using electrospinning [94].

Poly(3-alkylthiophene)s (P3ATs) are one of the most important types of PTs because they present high electrical and thermal conductivity, processability, and environmental stability [95]. However, P3AT has a high price; thus, its practical applications are limited [96]. Within the P3AT family, regioregular poly (3-hexylthiophene) (P3HT), poly(3-pentylthiophene) (P3PT), and poly(3-butylthiophene) (P3BT) are well-known CPs that have been widely used in organic electronic sensing applications owing to a good balance between the solubility and electric properties. With the increase of alkyl chains, the degree of phase separation is gradually improved and a balanced hole/electron transport enables to be achieved [90,97,98,99].

### 2.6. Poly(o-phenylenediamine) (PoPDA)

PoPDA is recently considered a low-cost electroactive organic material and an attractive CP widely used in sensors, energy-conversion devices, and biomedical fields [100]. It exhibits good solubility, processibility, high electroactivity, and thermal stability [101]. PoPDA has a ladder-type CP with a phenazine-like structure, in which an o-phenylenediamine, an aniline derivative with an amino group at its ortho position, is polymerized in an aqueous hydrochloric acid medium (pH < 1) [102]. The molecular properties of PoPDA are remarkably different from PANI, which contains a 2,3-diaminophenazine repeat unit in a ladder-like fashion or an open PANI-like structure [103]. To enhance spectral, morphological, and photo physical properties, PoPDA can be doped by luminol to form luminol-doped PoPDA.

PoPDA can be prepared by similar methods to PANI, including (i) using an oxidizer to polymerize o-phenylenediamine at room temperature [104]; (ii) using a reprecipitation approach to prepare nano/micro-structured PoPDA by transferring the o-phenylenediamine from a “good” solvent to a “bad” solvent where the o-phenylenediamine precipitate due to its lower solubility. For the chemical oxidation method, using high redox potential reagents, i.e., FeCl_3_, AgNO_3_, HAuCl_4_, and (NH_4_)_2_S_2_O_8,_ is required to form PoPDA structures. In the reprecipitation procedure, the formation of 1D structures is facilitated when transferring o-phenylenediamine from N-methyl pyrrolidone to water [105]. For the preparation of PoPDA nanofibers, direct electrospinning has been reported as a potential process [106].

**Table 1:** Summarized structures, properties, synthesis methods, and applications of CPs for flexible devices.

CPs	Structures	Synthesis methods	Properties	Refs
**PA**	-Linear polyene chain and multifaceted properties.	Ziegler−Natta catalysis, non-catalytic polymerization, and precursor-assisted synthesis.	Good electrical conductivity, photoconductivity, gas permeability, supra-molecular assembly formation, chiral recognition, helical graphitic nanofiber formation, and liquid crystallization capability.	[15,16,24,27,28]
**PANI**	Three different forms: leucoemeraldine, emeraldine, and pernigraniline.	Chemical oxidation method, interfacial polymerization, and electrospinning.	Leucoemeraldine (insulator), pernigraniline (insulator), and emeraldine salt (10^−2^–10^0^ S/cm)	[30,32,52,107]
**PEDOT**	A monoclinic lamellar structure consisting of inclined π-stacks.	Chemical polymerization, electrochemical polymerization, and electrospinning.	-The highest conductivity of 6259 S.cm^−1^ for thin films and 8797 S.cm^−1^ for single crystals. -Ultra-low thermal conductivity, processability, non-toxicity and unique flexibility, water insolubility.	[53,54,56,59]
**PEDOT:PSS**	PEDOT: PSS grains with a nano-size (30–50 nm).	Chemical polymerization, electrochemical polymerization, and electrospinning.	High conductivity, air stability, transparency, flexibility and intrinsic stretchability, and water insolubility.	[61,63,64,65,68]
**PPy**	A heterocyclic and positively charged CP.	Electro-polymerization, vapor-phase polymerization, electrospinning, microemulsion polymerization.	Good environmental stability, high conductivity, and good redox properties.	[76,77,78,108]
**PT**	Four different oligomeric structures (2-ring, 4-ring, 6-ring, and 8-ring).	Chemical oxidation, electrochemical oxidation, oxidative chemical vapor deposition, and electrospinning.	High stability, structural modification, and solution processability.	[91,92,94,109]
**PoPDA**	A ladder polymer possessing a phenazine-like structure.	Chemical oxidation, electrochemical polymerization, electrospinning, and reprecipitation.	Good solubility, processibility, high electroactivity, and thermal stability.	[101,104,105,106]

## 3. Advanced Techniques for CP Deposition on Flexible Devices

### 3.1. Solution-Based Methods

**Figure 1:**
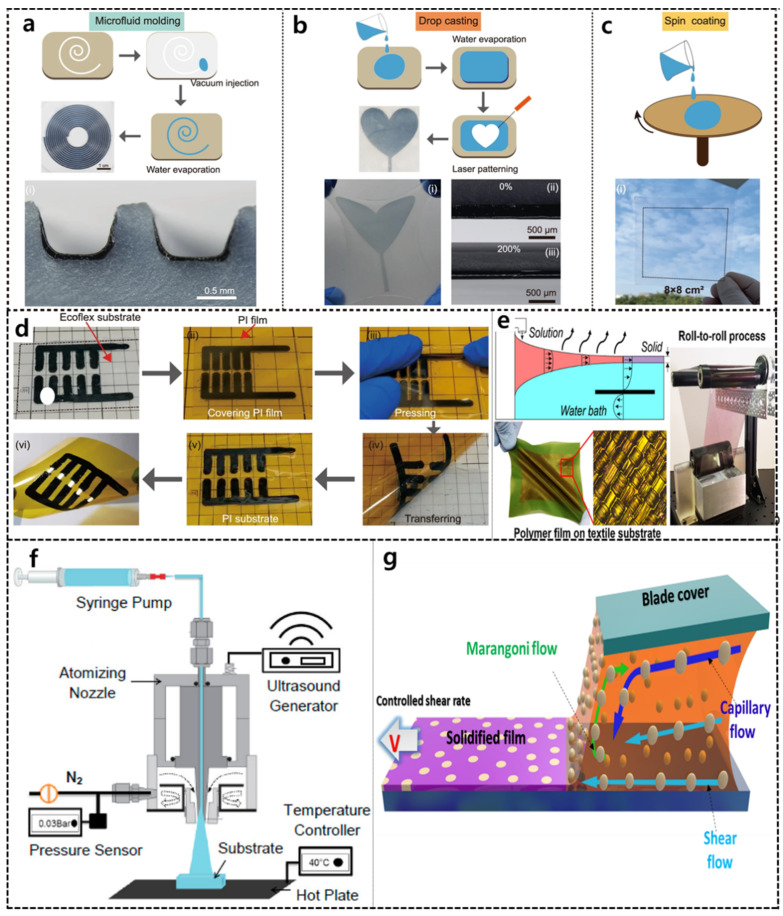
Some solution-based methods for the preparation of CP-based flexible devices: (**a**) Microfluid molding, (**b**) drop casting, (**c**) spin coating, (**d**) combination of drop casting and laser patterning (Reproduced with permission from ref [112]); (**e**) roll-to-roll coating of CP film onto a plastic substrate (Reproduced with permission from ref [121]); (**f**) spray-coating system for CP deposition (Reproduced with permission from ref [124]); (**g**) shear coating system (Reproduced with permission from ref [109]).

Solution-based techniques for depositing CPs are the simplest and lowest-cost technologies for developing and designing flexible devices. In this set of approaches, the CP molecular crystallinity can be controlled by using high-boiling-point solvents or blends of good and poor solvents or by controlling physical force effects such as centrifugal, shear, and capillary forces [110]. In recent decades, various solution-processing techniques (i.e., spin coating, drop casting, bar coating, solution shearing, and printing) have been used for depositing CPs on flexible devices owing to their excellent stability, homogeneity, and ability to produce patterns without the “coffee ring” effect [109,111]. Figure 1a–c shows the microfluid molding, drop-casting, and spin-coating methods [112]. In microfluid molding, CP films are deposited in microchannels, yielding special U-shaped films on the microchannel walls (Figure 1a). The drop-casting technique involves the formation of CP thin films by first dropping a CP solution onto a flexible substrate, followed by solvent evaporation (Figure 1b). In this technique, the long duration and slow assembly of the CP film can improve the film crystallinity; however, the final films have low uniformity and many vacancies. Thus, drop-casting and laser-patterning techniques enable the fabrication of patterned flexible devices with high transparency (Figure 1d) [112]. In addition, plasma technology has been employed as an adhesion promoter between the polyethylene terephthalate (PET) substrate and the CP layer to improve the drop-casting technique [113]. Recently, Luo et al. developed a capillary action strategy to mediate CP chain self-assembly during drop-casting [114]. In this approach, a sandwich tunnel system was designed using functionalized glass spacers that induced capillary action to control the nanostructure, crystallinity, and charge transport properties of the CP films.

Spin coating is the most common technique in widespread use for fabricating and depositing CPs in flexible devices because of its simplicity, speed, and low cost (Figure 1c) [112]. In spin-coated devices, a polydimethylsiloxane (PDMS) encapsulation layer is often spin-coated layer by layer on the PET substrate. Then CP films are deposited on the substrate by spin-coating. However, the spin-coating method has several drawbacks, including material wastage, restriction to small-scale substrates, uncontrollable crystallinity, and high film thickness. Modifying the spin-coating method has been developed to save materials and obtain ultrathin films [115,116]. A simple spin-coating approach with on-the-fly dispensing was successfully developed to prepare ultrathin films (≤10 nm) [115]. In this method, the spin-coater motor uses a high rotation rate during solution drop-ping, which results in rapid solution spreading, and the static wetting balance on the hydrophobic substrate is broken by the tangential force. A novel off-center spin-coating method was also introduced to allow the growth of a highly aligned metastable structure of a blended solution [116]. In this spin-coating technique, the solution is dropped on the substrate away from the center of the spin coater. The centrifugal force can induce high orientation and phase separation in the film, leading to high CP mobility.

Dip coating is a simple, well-established, waste-free, low-cost, and low-energy consumption method and is a relatively common technique used both in laboratories and industrially [117]. This technique has three main steps: (i) the substrate is immersed in the CP solution for a sufficient time, (ii) a CP thin film covers the substrate while it is pulled up from the solution, and (iii) the dry CP thin film forms after solvent evaporation [118,119]. The film thickness in dip coating can be easily controlled by adjusting the CP solution viscosity and withdrawal speed [120]. However, obtaining homogeneous and ultrathin (<20 nm) films is difficult. To develop flexible devices, the roll-to-roll dip-coating process is often used, in which flexible substrates (e.g., polyimide film and PET) are used with a bath of low-viscosity CP solution. The flexible substrate is dipped and pulled out using a rotating roll (Figure 1e) [121]. With this process, the film thickness can be precisely modulated in the ultrathin range (10–80 nm) by varying the velocity and tuning the concentration of the CP solution, allowing the formation of ultrathin films with a length of >1 m and an area of 1000 cm^2^. Moreover, the roll-to-roll dip-coating technique can be used to prepare films with decreased elastic moduli and improved extensibility, which are necessary mechanical properties for stretchable and flexible devices. However, the process requires a large solution storage tank; thus, volatile and low-boiling-point solvents cannot be used.

Spray coating is a convenient, cost-effective, and industrially scalable deposition technique suitable for depositing CPs on 3D surfaces during low-temperature processing to create electronics [122,123]. A spray nozzle is set perpendicular to a flexible substrate, and the CP solution is directly sprayed onto the substrate, where it can form a very thin, dense, and stable film (Figure 1f) [124]. In general, the atomization process, in which tiny droplets are formed by the passage of the CP solution through the narrow orifice of the spray nozzle, is controlled by N_2_ air pressure [125] or an ultrasonic technique [126]. Ultrasonic spray coating is preferred owing to its simplicity, good transfer efficiency, cost-effectiveness, small droplets, and good reproducibility [127]. Several parameters can be manipulated to control the surface morphology of the film, including the air pressure, viscosity, solvent properties, N_2_ pressure, the distance between the nozzle and substrate, spray time, and spray temperature. It has been demonstrated that the spray coating method can produce high-quality CP thin films on flexible and 3D substrates [123]. An alternating spray deposition method was recently successfully developed to fabricate multicomponent films with distinct material properties from independent sources [128]. This method is based on the partial coverage of alternating donor and acceptor layers to construct an interpenetrating network of nanodomains, mimicking layer-by-layer deposition. Thus, alternating spray deposition can be applied to various material systems.

The solution shear-coating method is a simple, efficient, low-cost, and scalable technique for preparing oriented CP thin films for flexible devices [129]. In the solution-shearing process, the CP solution is dropped on a heated substrate, followed by dragging a shearing plate, and the CP thin film is obtained after solvent evaporation (Figure 1g) [109]. Owing to the interplay of the viscous force, capillary force, and Marangoni flow, the crystallinity, uniformity, and morphological features (i.e., pore size, pore shape, and film thickness) of CP films can be controlled [109]. This technique can be used to prepare nanoporous ultrathin films with pore sizes and thicknesses in the nanoscale range and to increase the charge-carrier mobilities of CP films because of the greater electron orbital overlap between the component molecules. Shear coating possesses several advantages, including (i) the CP concentration remains constant over a wide range of temperatures and coating times; and (ii) small amounts of CPs are used (~20 mL·cm^−2^ of the substrate) [130]. Thus, the shear coating can be used to develop high-performance, large-scale, low-cost CP flexible devices [131]. However, the technique remains limited by the required substrate surface, the surface roughness of the obtained CP films, and device-to-device variability.

Several strategies have been developed to improve the performance of CP thin films obtained from solution-based deposition methods, such as the selection of appropriate solvents and the pretreatment of substrates [123]. Solvent type is a critical factor for the morphological formation and crystallization of CP thin films. A solvent with a higher boiling point induces a lower crystallization rate because the CP molecules self-organize into larger crystal domains after the longer time available before evaporation. To use high-boiling-point solvents, the substrate must be heated to optimize the deposition efficiency of CP thin films. However, the increased temperature could cause a Marangoni flow from the high-temperature zone (bottom) to the low-temperature zone (top), which might yield nonuniform CP thin films. To overcome this problem, a blended solvent system creates a countercurrent flow, compensating for the loss of molecules induced by the Marangoni flow [132].

**Figure 2:**
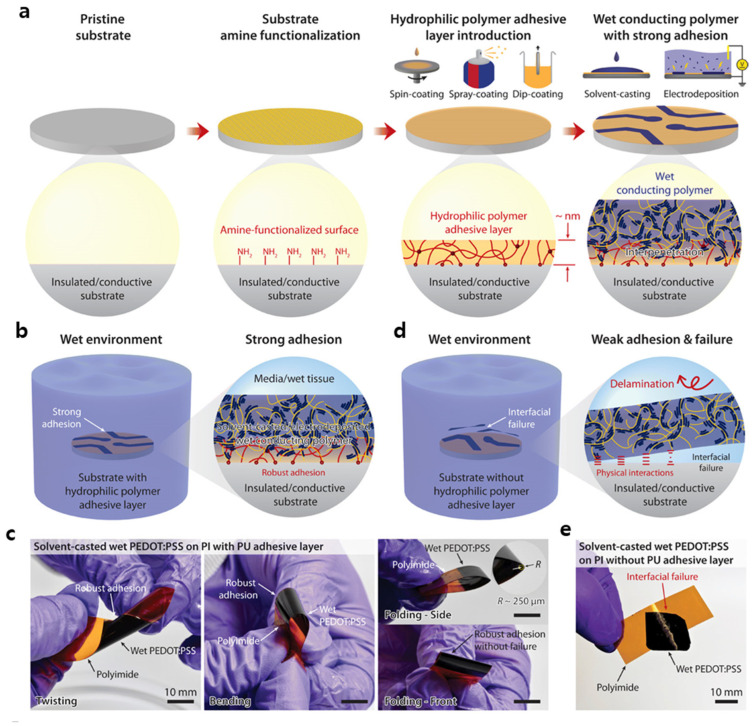
Strong adhesion of CP on different substrates. (**a**) An amine-functionalized substrate with a hydrophilic polymer adhesive layer, (**b**) A substrate with an adhesive layer in a wet environment, (**c**) A polyimide substrate with a polyurethane adhesive layer, (**d**) Weak and unstable adhesion without the adhesive layer, and (**e**) A polyimide substrate without the adhesive layer (Reproduced with permission from ref [134]).

The weak and unstable adhesion of CPs to substrates can lead to CP debonding or reduced reliability and efficacy of flexible devices [133]. Some methods have been reported to enhance CP adhesion, including the topological modification of substrates and electrodeposition. To achieve strong adhesion in flexible devices, a simple method has recently been applied to various wet CPs, including PE-DOT:PSS, PPy, and PANI, on diverse, flexible substrates, including glass, polyimide, PDMS, and indium tin oxide (ITO) (Figure 2) [134]. In this method, a hydrophilic polymer adhesive layer with nanoscale thickness was introduced for the pretreatment of the substrate; this layer not only provided strong substrate adhesion but also penetrated the polymer network. In addition to utilizing a broad range of substrate materials, such approaches can be implemented using different coating techniques. The results have indicated that owing to the strong and stable interfacial adhesion between CPs and different substrates, this method can be utilized for commercial CPs and is compatible with various other fabrication techniques, such as solvent casting and electrodeposition.

### 3.2. Printing Technologies

Currently, printing technologies are commonly used for depositing CPs in the fabrication of electronic devices. Compared to the above solution-based methods, these techniques offer several advantages, such as saving materials, depositing on large-area substrates, and accurate patterning. Many types of printing technologies can be used for deposing CPs; inkjet printing, screen printing, and 3D printing are widely employed for stretchable and flexible devices.

#### 3.2.1. Inkjet Printing

**Figure 3:**
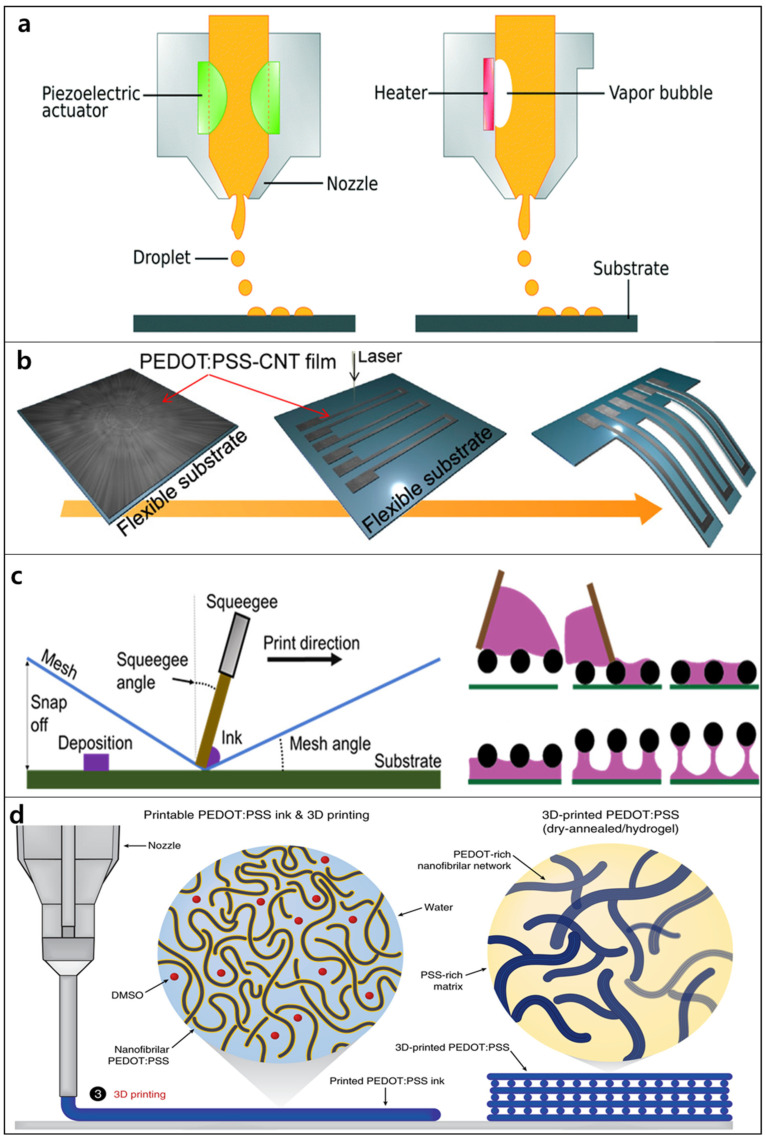
Schematics of (**a**) the inkjet printing process: piezoelectric mode and thermal mode (Reproduced with permission from ref [137]); (**b**) preparation of PEDOT:PSS/CNT film-based artificial electronic whisker using inkjet printing (Reproduced with permission from ref [145]); (**c**) the ink transfer process from screen to substrate and the three stages of screen printing (Reproduced with permission from ref [147]); (**d**) fabrication of a 3D-printable conducting polymer ink (Reproduced with permission from ref [155]).

Inkjet printing sometimes termed “direct-writing,“ is one of the most important technologies for designing highly stretchable and flexible devices owing to its precise deposition in well-defined patterns of picoliter-scale volumes of CP solutions [135]. The translation stages and ink dispensers used in inkjet printing can be controlled by a computer, facilitating the production of complex patterns. Due to this method’s mask- and contact-free direct-writing ability, inkjet printing decreases cost and CP material use, reduces waste, and minimizes contamination. In this method, an ink containing a CP solution is transported from the ink reservoir to the nozzle head, and an ejecting process then forms microdroplets. The printing step is performed as the ink droplets collide with the substrate at specific rates [136]. Printed patterns can be obtained by controlling the displacement of the substrate or the printhead, followed by evaporation and solidification. In general, the printed substrate requires high-temperature post-processing treatment, such as thermal annealing, sintering, or calcination, to remove solvents and increase adhesion. Inkjet printers can operate in two main modes: continuous or drop-on-demand (DOD) [137]. In continuous inkjet mode, the ink suspension is ejected from the nozzle as a liquid jet that is broken up into droplets by surface tension [138]. The DOD inkjet mode is commonly used for designing flexible devices owing to its high precision, small drop size, and flexibility [139]. The DOD mode often uses either piezoelectric or thermal actuators (Figure 3a) [137]. In DOD printing with piezoelectric actuators, an electric field induces the deformation of the piezoelectric actuator, which results in the formation and release of ink droplets. At a certain electric potential, the piezoelectric transducer causes a pressure wave that moves towards the nozzle and drives out the CP ink droplet. In DOD printing with thermal actuators, a Joule heating element placed near the nozzle is used to heat the CP ink solution and form bubbles of ink vapor, followed by routing out ink micro-droplets [138]. Because evaporation is used to create the ink droplet, the ink selected in this approach must have volatile components [138,140].

The inkjet printing technique has been successfully applied to develop and fabricate numerous CP-based flexible devices [12,141,142,143]. For instance, Wang et al. introduced electronic devices featuring micrometer-sized patterns of highly stretchable and transparent PEDOT:PSS using inkjet printing [12]. The high stretchability of PEDOT:PSS films requires the use of enhancers to create soft domains and achieve high fracture strain. The study by Wang et al. indicated that highly stretchable and conductive PEDOT films with high cycling stability could be obtained by incorporating various ionic additives, such as dioctyl sulfosuccinate sodium salt, sodium dodecylbenzenesulfonate, dodecylbenzenesulfonic acid, and ionic liquids. The enhanced conductivity and stretchability of the CP films were ascribed to a synergistic effect, in which the ionic additives can both have good solubility (in water and the PEDOT:PSS matrix) and function as effective dopants for PEDOT molecules owing to the highly acidic anions. Recently, the use of inkjet printing technologies in the development and design of flexible electronic devices has attracted considerable interest in flexible electronic skin (e-skin) platforms that can operate at low temperatures [141,144,145]. For instance, a printed high-sensitivity multifunctional artificial electronic whisker (e-whisker) sensor was successfully fabricated using printable nanocomposite PEDOT:PSS inks (Figure 3b) [145]. Moreover, multifunctional e-whisker arrays were developed by mapping 2D and 3D distributions based on inkjet printing.

#### 3.2.2. Screen Printing

Screen printing has been considered a promising industrial deposition technique that can be scaled up by sheet-by-sheet or roll-to-roll processing [146]. In the screen-printing method, an ink suspension is directly transferred from a stenciled mesh to a target substrate (Figure 3c) [147]. The printing mechanism has three stages [148]: (i) the CP ink solution is flooded into a mesh; (ii) this mesh contacts the substrate under a downward force and the ink adheres to the substrate and the mesh owing to the interface free energy; (iii) the ink is deposited on the substrate by vertically pulling the mesh upward. This technique can produce pattern resolutions of at least 100 μm for various shapes and sizes within seconds. Moreover, owing to its over-printability, highly accurate alignment, registration marks, layer-by-layer deposition, and suitability for mass production, screen printing has been used extensively for the preparation of CP-based flexible devices [149,150,151]. However, the screen-printing process must meet four essential requirements [152]: (i) the conductive ink must interact well with the stretchable target substrate interface; (ii) the highly conductive ink must maintain its conductivity with strain up to 20%; (iii) high stability must be maintained over multiple uses; and (iv) sufficient thickness is necessary to allow precise control of the fabrication of reliable and small features. In practice, screen printing has often been utilized in combination with vapor-phase polymerization to produce printed devices at high resolution with the possibility of over-printing; this provides commercial devices using a cost-effective deposition technique [146].

#### 3.2.3. 3D Printing

3D printing allows the fabrication of microscale structures in a programmable, facile, and flexible manner with freedom of design in 3D space, compared to other approaches [153,154]. In 3D printing, CP materials are deposited in a layer-by-layer manner using a movable nozzle, following a specific shape programmed by software (Figure 3d) [155]. Based on the method by which CPs are deposited, 3D printing follows one of two models: (i) fused-deposition modeling (FDM), which utilizes a melted filament of CP moved through a gear mechanism to a hot end, or (ii) direct ink writing (DIW), which uses a semi-melted CP filament that is deposited by the action of an applied current, air, pistons, or screws. 3D printing open a new level for the design and development of CP-based flexible devices because of its significantly reduced cost and ease of use [156]. However, the machines used in 3D printing can be more complex than conventionally machined parts. 3D printing has exhibited limited applicability of pristine CPs for specific 3D printers and optimized printing conditions. Moreover, to print and obtain the desired shape, the CPs used in both the FDM and DIW models must have specific rheological behaviors and viscosity values at different shear rates (<104 and 101 Pa·s at low and high shear rates, respectively) [157]. Thus, for 3D printing, most pristine CPs must be combined with other thermoplastics as composites or copolymers [158]. Furthermore, several factors must be optimized for printability when using CP composites, including the size and size distribution of CP fillers, printing temperature, printing speed, residence time, and printing bed temperature [156].

3D printing has been applied to fabricate stretchable CP electrodes for flexible and wearable devices. For instance, Yang et al. used the synergistic integration of carbon nanotubes (CNTs) and PEDOT:PSS to develop a stretchable electrode fabricated by DIW printing [159]. PEDOT:PSS/CNT composite ink was prepared by mixing PEDOT:PSS nanofibrils with a CNT suspension. This was freeze-dried to form a freestanding electrode. The device exhibited excellent deformability and flexibility, with a well-designed arc-shaped pattern. Yuk et al. developed a high-performance PEDOT:PSS ink for 3D printing based on a commercial PEDOT:PSS aqueous solution [155]. A simple method was used to endow this ink with the rheological properties of the pristine PEDOT:PSS solution, in which the 3D-printed CP structures could be converted into dry and hydrogel forms. PEDOT:PSS hydrogel ink has superior printability and allows the facile deposition of CP into high-resolution and high-aspect-ratio micro-structures.

## 4. Recent Applications of CP-Based Flexible Devices

CP-based flexible devices have been employed in various applications, including gas sensors, health-monitoring devices, strain and temperature sensors, and biosensors. Table 2 presents a summary of recent research on CP-based flexible sensors.

### 4.1. Gas Sensors

The development of flexible and wearable chemiresistive sensing devices has attracted significant attention. Due to their inherent flexibility and high conductivity, CPs are among the best candidates for designing such devices [160]. Moreover, CPs can work at room temperature, thus requiring minimal power and allowing their incorporation into small, flexible, wearable devices [161]. Therefore, several wearables, flexible, highly sensitive, and selective sensors have been successfully developed for various gases and vapors that are harmful to human health [162]. These devices were based on the incorporation of different CPs with flexible substrates, and they showed high sensitivity and selectivity for NH_3_ (5–1000 ppm) [163,164,165], NO [166], H_2_S [167], hydrazine [168], N_2_H_4_, CHCl_3_ [169], H_2_ [170], and volatile organic compounds (VOCs) [111]. In gas sensor devices based on CPs, the interaction of CPs and the gas analytes can be a chemical reaction or physical adsorption [171]. Chemical reactions can alter the doping levels and physical properties of the CP. Gases containing electron acceptors, such as NO_2_, I_2_, O_3_, and O_2_, are likely to oxidize CPs and increase their doping level because their electron affinities exceed those of the CPs. For example, the number of charge carriers in P3HT was found to increase after oxidative doping with NO_2_, thus reducing the resistance and increasing the conductivity of this CP [172]. PANI showed changes in its optical absorbance between 500 and 800 nm because of the oxidation and protonation of O_3_ gas. PANI and m-chloro-PANI both showed higher sensitivity than N-methyl-PANI due to the oxidation of its pernigraniline state [173]. In contrast, H_2_S, NH_3_, and N_2_H_4_ are electron-donating gases that de-dope CPs, resulting in increased resistance and reduced conductivity [109]. For non-reactive VOCs such as chloroform, acetone, aliphatic alcohols, benzene, and toluene, it has been supposed that weak physical interactions of CPs with these analytes can modify the CP resistance and conductivity: (i) ethanol and hexanol may be absorbed on dipentoxy-substituted polyterthiophene, changing the potential barrier of polyterthiophene grains [111]; (ii) swelling behavior increases the distance between PANI and PPy chains, leading to increased resistance when exposed to and absorbing chloroform, acetone, ethanol, acetonitrile, toluene, and hexane vapors [169]; and (iii) the diffusion of acetone into PPy intersegmental spaces damages the interactions between aromatic pyrrole rings, resulting in increased disorder and decreased conductivity [174]. In summary, portable, low-cost, and flexible CP-based chemiresistor devices have been designed and shown promising applications in the detection of numerous gases and vapors with high sensitivity and selectivity at low concentrations and fast response times.

**Figure 4:**
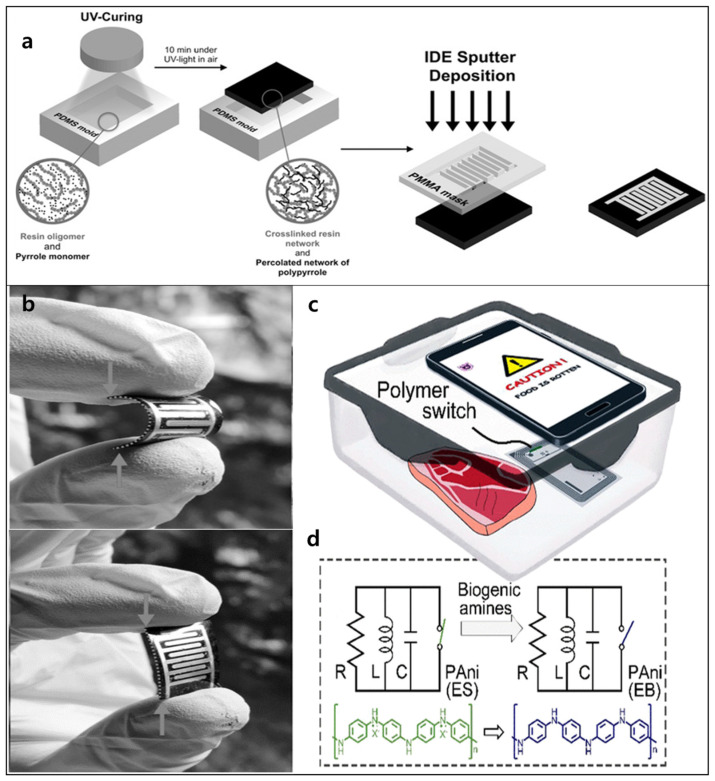
(**a**) Schematic of UV light fabricating process and (**b**) image of a flexible PPy humidity sensor (Reproduced with permission from reference [178]). (**c**) The wireless badge for food spoilage detection based on PANI thin film and (**d**) the circuit of the modified NFC tag for detection of gases released from spoiled meat (Reproduced with permission from reference [183]).

Respiration monitoring, particularly via humidity sensing, is an important and easy approach to evaluating human health [175]. Human exhaled breath generally has a high relative humidity (>90%) that does not change under the impact of movement, environment, or season. Compared to other materials, CPs exhibit several advantages in the design of humidity sensors, including their easily modulated structures, facile processing, capacity for large-scale fabrication, and low cost. However, in humidity sensors for respiration, the materials must satisfy two major conditions: (i) high stability under long-term exposure to high-humidity conditions and (ii) fast response time (~1 s) due to the short time of a single respiratory cycle (3–4 s). Therefore, CPs are often modified or blended with other materials in composites to enhance their characteristics to suitable levels for flexible humidity sensors. It has been demonstrated that PANI and PPy show relatively good sensitivity to humidity due to the “proton effect” and their changed conductivities in the presence of H_2_O vapor [176]. Guo et al. developed composites of PANI nanofibers with spandex-covered yarns, which showed high stretchability and conductivity [177]. Owing to the unique spiral configuration and preservation of large prestrain, the use of the spandex-covered yarn resulted in a PANI nanofiber composite with stable conductivity at different elongations (even stretching up to 200%) and excellent cyclic performance. The stretchable humidity sensor based on the PANI nanofiber composite exhibited excellent sensing with good sensitivity, fast response, and good repeatability. In flexible humidity sensors based on CPs, expensive fabrication is a significant limitation for practical applications. To overcome this problem, a one-step method for the fabrication of flexible PPy humidity sensors was developed using simultaneous UV curing and in-situ photopolymerization [178]. In the synthesis process (Figure 4a), PPy polymerization can be achieved by the UV irradiation of monomers/oligomers. Flexible PPy films are then obtained by interpenetrating the insulating network with different flexible substrates (Figure 4b). This flexible PPy sensor showed remarkable sensing capabilities, making it a promising alternative to other humidity sensors owing to its printability, flexibility, and simple one-step fabrication.

Precise, convenient, and low-cost technology for monitoring and evaluating the status of food to avoid spoilage has gained great interest for consumers, storage purposes, and supply chains. In general, monitoring methods for food spoilage, especially that of meat, focus on the detection of total volatile basic nitrogen, including biogenic amines (BAs) and ammonia (NH_3_) [179]. BAs, such as putrescine and cadaverine, cause the bad odors of meat decomposition and are mainly formed through the decarboxylation of amino acids upon interaction with microbes; they are considered the most significant markers of meat spoilage [180]. However, conventional methods (i.e., chromatography, electrochemistry, chemiluminescence, and spectrometry) currently used to detect BAs [181] have highly limited practical applicability because of the shortcomings of bulky and expensive equipment, poor portability, and complex sample processing. Therefore, flexible chemiresistive gas sensors based on CPs are considered promising alternatives for determining BAs in foods [182]. Ma et al. developed a nanostructured PANI-based gas sensor with high sensitivity to the gases emitted from meat decomposition (Figure 4c) [183]. The PANI-based sensor was incorporated into the circuit of a near-field communication (NFC) tag with a sensitive switch to detect food spoilage using a smartphone (Figure 4d). It has been suggested that films of PANI and other CPs can be broadly employed to design intelligent sensors for food status monitoring applications in daily life, storage, and supply chains.

### 4.2. Strain Sensors

**Figure 5:**
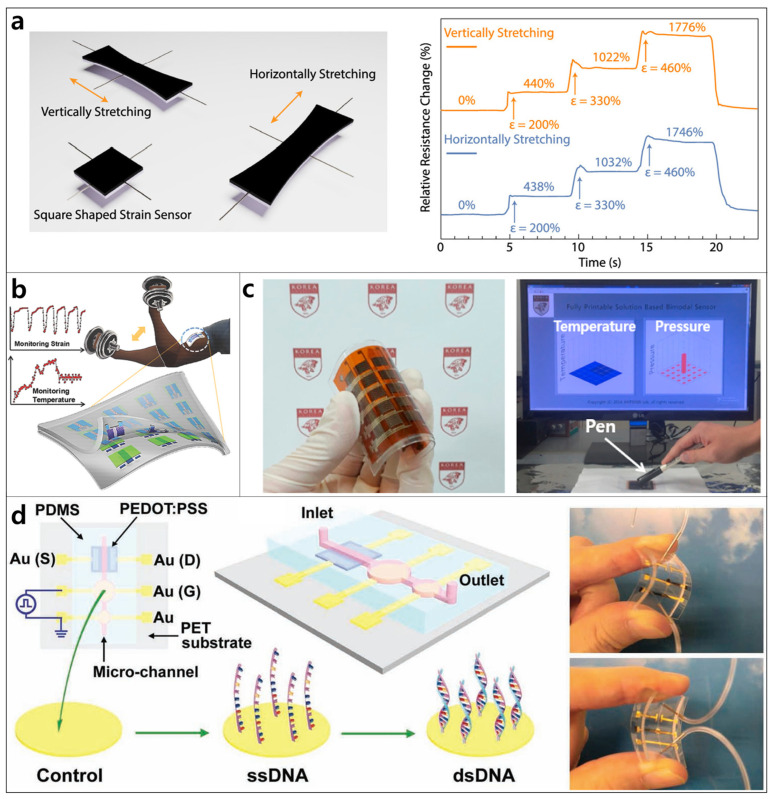
(**a**) Schematic demonstrating omnidirectional sensing capability and relative resistance change over time with the horizontal and vertical tensile stretching of a PANI-based strain sensor (Reproduced with permission from reference [188]); (**b**) schematic of a transparent, stretchable gated sensor array for strain and temperature detection (Reproduced with permission from reference [196]); (**c**) photograph of a flexible sensor array for measurement of pressure and temperature by touching the sensor with a pen (Reproduced with permission from reference [141]); (**d**) an OECT flexible biosensor integrated with a microfluidic system for DNA detection (Reproduced with permission from reference [214]).

Wearable and flexible strain sensors are essential components for various applications, such as healthcare monitoring, soft robots, and immersive gaming. These flexible sensing devices must have comfortable skin adhesion, high-accuracy monitoring capacity for human motion, and excellent durability. For the design of such strain sensors, the materials used must possess some unique features, including superior stretchability, excellent flexibility, a wide sensing range, high sensitivity, and the possibility of coating on elastomers, that is, PDMS and rubbers. Compared with metals or other semiconductors, CPs show several prominent advantages in stretchability, flexibility, environmental stability, and processability, making them suitable candidates for fabricating flexible and stretchable strain sensors. In addition to their high stretchability and good flexibility, CP-based flexible and stretchable strain sensors have recently attracted significant attention owing to their excellent durability, tunable strain-sensing behavior, and ease of processing [184]. Depending on the sensing mechanism, the efficiency of CP-based strain sensors can be influenced by factors including the CP nanoparticle shape and size dispersion, synthesis method, and interactions of CP particles with other polymer molecules [185]. Considering the above CP advantages, many studies have successfully developed CP-based strain sensors with good performance, wide workable strain ranges, high sensitivity, and good repeatability [186,187]. For example, a wearable PANI-based strain sensor that exhibited ultrahigh stretchability (1935%), good repeatability (>98%), and high responsivity to both strain (R > 0.9998) and flexion bending (R > 0.9994) was recently fabricated (Figure 5a) [188]. This sensor also possessed skin compliance, elasticity, and self-healing ability. Furthermore, PANI and other CPs can be scaled and processed in an environmentally friendly manner; thus, CPs have high potential utility for developing next-generation flexible strain sensors.

For practical application, flexible strain sensors must be fabricated economically on macroscale substrates. Mammalian-mimicking functional electrical devices have demonstrated great potential as technologies in robotics, wearable and health-monitoring systems, and human interfaces owing to the following advantages: (i) economical fabrication of macroscale devices on flexible substrates, (ii) high sensitivity, and (iii) multifunctionality. Recently, e-whiskers have been designed to detect strains effectively [189]. Based on these factors, Harada et al. introduced a fully printed multifunctional PE-DOT:PSS e-whisker array with high strain-sensing performance [145]. The e-whisker arrays were patterned by screen printing, which can be developed as an economical macroscale fabrication technique. In addition, the e-whisker arrays exhibited multifunctional ability by successfully mapping 2D and 3D distributions of the arrays. CP-based e-whisker arrays designed as fully printed, macroscale multifunctional devices are likely to be considered for developing low-cost, highly flexible, and stretchable electronics and robotics for strain detection.

### 4.3. Temperature Sensors

The accurate monitoring of physiological factors, particularly temperature, is critical in disease diagnosis and tracking of various medical conditions [190]. Developing flexible and stretchable sensors is a potential approach for enhancing the efficiency of monitoring dynamic and spatial variations in temperature. CPs are considered promising materials for designing and developing highly effective sensors with excellent flexibility and temperature sensitivity [191,192]. Another advantage of CPs over other materials in the design of temperature sensors is their ability to form patterns on various substrates by printing techniques without using photolithography. In temperature sensors, CPs may experience expansion and the separation of CP particles as the temperature increases, resulting in increased resistance, indicating a positive temperature coefficient. To improve the stimuli-response properties of CPs in flexible temperature sensors, CPs have been incorporated with other materials in composites to obtain better electrical properties upon exposure to the stimuli of interest [192,193]. A CP/reduced graphene oxide (rGO) composite-based temperature sensor was de-signed by patterning a composite on a flexible and stretchable substrate [194]. The sensor showed a temperature coefficient of resistance with a high responsivity of 0.008/°C, as well as good selectivity to temperature with respect to pressure and moisture. Such stretchable devices with meandering patterns have also demonstrated potential as healthcare-monitoring devices.

Developing transparent and stretchable temperature sensors based on gated structures and all-elastomeric materials simplifies the fabrication process while achieving a high yield and low cost. However, intrinsically transparent and stretchable devices containing multiple parts, that is, electrodes, gate dielectrics, active layers, and sensing materials, remain difficult to fabricate [195]. Thus, intrinsically transparent temperature sensors based on novel materials with high transparency and stretchability are promising. Trung et al. successfully developed an all-elastomeric transparent, stretchable gated temperature sensor array (Figure 5b) [196]. This temperature sensor array was further integrated with a strain sensor, yielding a platform that offers easy attachment to the human skin. Moreover, this sensor device makes novel use of a CP composite, which is intrinsically transparent, stretchable, and easily coated directly onto the transparent, stretchable substrate. The sensor showed high stretchability (a strain of 70%), resistance sensitivity to temperature (~1.34%/°C), and good stability (10,000 cycles). In recent years, flexible sensors have been fabricated using CP-based composite materials with skin-like mechanical properties [197]. Incorporating CPs with carbon materials such as graphene and its derivatives into conductive hydrogels has been a common and facile strategy for fabricating highly sensitive and conductive flexible temperature sensors [198,199]. For instance, sandwich-like PPy–rGO–PPy nanostructure-based gelatin hydrogels were prepared by a universal interface method, in which rGO and pyrrole monomers were polymerized at 100 °C [200]. This skin-like sensor showed excellent mechanical and electrical behavior with high sensitivity for detecting the temperature and motion of the human body. Similarly, e-skin, which mimics human skin functions in its flexibility and ability to sense pressure and temperature gradients, can be implemented in intelligent robots and next-generation wearable pressure and temperature sensors [201,202]. Kim et al. introduced a flexible sensor for e-skin applications that simultaneously detected pressure and temperature (Figure 5c) [141]. The sensor was constructed by the inkjet printing of a PEDOT:PSS/AgNP composite on silicone rubber or an elastomer substrate. Such a sensor with a 25-pixel bimodal sensor array exhibited a high sensitivity to temperature (0.32%/°C with the small temperature change of 0.5 °C).

### 4.4. Biosensors

Flexible and stretchable biosensors for point-of-care (POC) testing systems have become emergent technologies with potential applicability in customized personal health monitoring systems for the early detection and control of diseases [203,204,205]. Current strategies for developing flexible and stretchable biosensors have focused on synthesizing advanced materials with multifunctional properties that can operate as sensing elements [206,207]. Among these materials, CPs are candidates for fabricating flexible biosensors; they have been utilized as bioreceptors to detect different biomarkers [208,209]. Hence, the design and development of CP-based flexible and wearable biosensors may become one of the most vital strategies for the early detection of human diseases in the future, especially considering the pandemic of coronavirus disease [210].

The key CP-based flexible electronic biosensors include organic field-effect transistors (OFETs) and organic electrochemical transistors (OECTs), owing to their miniaturization, low cost, intrinsic flexibility, tunable conductivity, biocompatibility, and mass producibility [211]. Based on their properties, PPy, PANI, and PEDOT are widely used in flexible biosensors [206,207]. PEDOT:PSS is often employed as a CP active channel material in flexible OECT bio-sensors for detecting bacteria [212], cancer biomarkers [213], glucose [208], and DNA/RNA [214]. PEDOT:PSS flexible biosensors can be fabricated by incorporating OECTs into flexible microfluidic systems. For example, a flexible DNA biosensor was developed by integrating a flexible microfluidic system with an OECT consisting of a PE-DOT:PSS active layer and Au gate electrode (Figure 5d) [214]. The OECT was first obtained by patterning on a flexible PET substrate, which was then integrated with a PDMS-based microfluidic device on the top. Such flexible biosensors show high flexibility and excellent sensing performance with a low limit of detection (LOD) (1 nM). Therefore, CP-based OECTs in combination with microfluidic systems are a promising strategy for the fabrication of flexible, highly sensitive, low-cost, and disposable biosensors.

CP hydrogel-based devices have recently been considered ideal candidates for designing flexible biosensors for POC systems [215]. A robust and force-sensitive CP hydrogel was prepared by polymerizing polyacrylamide and PANI into swollen chitosan microspheres [216]. Owing to the novel microsphere structure, the stretchability and mechanical stability of the hydrogel system were enhanced up to a strain of 600%, and the hydrogel system was used for flexible and wearable biosensors. The PANI hydrogel microspheres exhibited a rapid response and long-term electrical stability, indicating their applicability for developing flexible and wearable biosensors.

**Table 2:** Summary of recent applications of conductive polymer-based flexible sensors.

CPs	Applications	Substrate	Response	LOD	Response Time	Refs
**Gas sensors**						
PEDOT:PSS	H_2_ sensor	Substrate-free	31.6%	0.25% H_2_	19s	[170]
PPy	NH_3_ sensor	PET	39.4%	10 ppb	36 s	[171]
PANI	Humidity sensing	Paper	99.2%	5%	18 s	[176]
PANI	Humidity sensing	Substrate-free	90%	20%	30 s	[177]
PPy	Humidity sensing	PDMS	180%	20%	4000 s	[178]
PANI	Food spoilage detection	-	225%	5 ppm NH_3_	112 s	[183]
PPy	n-butylamine gas sensor	Copper interdigital electrode	8%	0.42 ppm	-	[217]
P3HT	NO_2_ sensor	Si/SiO_2_ wafer	98.98	10 ppm	100 s	[218]
PEDOT:PSS	H_2_S sensor	PET	62.4%	100 ppm	-	[219]
PPy	Dimethyl methylphosphonate sensor	Substrate-free	28%	0.1 ppb	100 s	[220]
PA	Food spoilage detection	Substrate-free	Changed color	100 ppm	10 min	[221]
P3BT	NH_3_ sensor	PET	17%	1 ppm	20 s	[222]
P3HT	NO_2_ sensor	Silicone wafer	30%	5 ppm	1 min	[223]
PoPDA	Humidity sensing	-	75%	11%	200 s	[224]
**Strain sensors**						
PEDOT:PSS	Monitoring starch-based food processing	Substrate-free	300%	-	50min	[186]
PANI	Strain sensor	Substrate-free	640%	100% strain	3 s	[188]
PEDOT:PSS	Monitoring human motions	PDMS	170%	1.6% strain	2 s	[225]
PANI	Monitoring physical motions	Substrate-free	296%	1% strain	2 s	[226]
PANI	Monitoring pulse waves	Substrate-free	150%	1% strain	20 s	[227]
PANI	Detecting finger motion	Poly(vinylidene fluoride) membrane	25%	5.2% strain	40 s	[228]
PPy	Human breath detection	Polyurethane substrate	35%	10% elongation	-	[229]
PPy	Pressure sensor	PDMS	240%	5 kPa stress	1.3 s	[230]
PPy	Human motion detection	A filter paper	135%	30% strain	5 s	[231]
PEDOT:PSS	Strain-temperature dual sensor	PVA substrate	6%	1% strain	-	[232]
P3HT	Stretchable strain sensors	PDMS	50%	10% strain	20 s	[233]
**Temperature sensors**						
PEDOT:PSS	Monitor surface temperature of human body	Polyurethane	1.34%/°C	22–38 °C	90 s	[196]
PEDOT:PSS	Detecting skin temperature	Polyimide substrate	0.03%/°C	30–45 °C	1 s	[199]
PPy	Detecting skin temperature	Substrate-free	1.288%/°C	35–40 °C	20 s	[200]
PPy	Detecting skin temperature	Polyvinylidene fluoride membrane	5.76%/°C	24–48 °C	0.33 s	[234]
PEDOT:PSS/PANI	Sensing human body temperature	PET substrate	0.803%/°C	35–40 °C	200 ms	[235]
PANI	Temperature sensor arrays	PET substrate	1.0%/°C	15–45 °C	1.8 s	[236]
PANI	e-skin temperature sensor	PDMS substrate	456%	36.5–42 °C	-	[237]
PPy	Photothermal conversion and thermosensing applications	Nonwoven fabric	20%	32–35 °C	30 s	[238]
P3HT	Photothermal sensor	Indium tin oxide	0.023 au/°C	25–75 °C	-	[239]
**Biosensors**						
PEDOT:PSS	DNA detection	Hydrogel substrate	-	17 fM	-	[240]
PANI	Hepatitis E virus detection	Glassy carbon electrode	500%	0.8 fg/mL	-	[241]
PPy	Detection of microRNA-21	Glassy carbon electrode	-	78 aM	-	[242]
PEDOT	Detection of biomarkers in human serum	Glassy carbon electrode	28%	35.64 mU/mL	30 min	[243]
PANI	Detection of *Escherichia coli* DNA	Screen printed electrode	-	4 CFU/mL	70 min	[244]
PEDOT:PSS	Cancer biosensor	Whatman filter paper	-	4 ng/mL	-	[245]
PEDOT/PEG	Detection of alpha-fetoprotein	Glassy carbon electrode	30%	0.0003 fg/mL	-	[246]
PEDOT:PSS	Detection of cellular electrical signals	Indium tin oxide	10%	10 µV	2s	[247]
PA	Detection of protein	Hydrogel	Changed color	20nM	60 min	[248]
PA	Detection of E. coli	Polyurethane	Changed color	9 × 10^8^ CFU/mL	0.5–3 min	[249]
PPy	Detection of Valproate	Screen-printed electrodes (SPEs)	8%	17.48 μM	8 min	[250]
P3HT	Protein biosensor	Glassy carbon electrode	2.3%	1 ng/mL	50 s	[251]
PoPDA	Cancer detection	Carbon electrode	-	8.4 × 10^−8^ ng/mL	8 min	[252]

## 5. Conclusions

Conductive polymers show flexibility in tuning their molecular structures and electrical and mechanical properties. They are readily solution-processable and patternable, allowing the fabrication of thin films on flexible substrates. Many solution-based processes have been used to prepare conductive polymer films; such methods offer advantages for the large-scale production of flexible devices. For optimization of the coating efficiency in solution-based methods, however, conductive polymers must be dissolved in appropriate solvents, and the substrate surface should be treated with additives to improve adhesion. Printing technologies such as inkjet printing, screen printing, and three-dimensional printing have been used as rapid, simple, flexible, low-cost, and high-resolution methods. These techniques can also save materials, deposit on large-area substrates, and achieve accurate patterning. With recent advances in ink chemistry, printing processes, and large-scale processability, printing technologies are considered promising for fabricating arrays of flexible sensors. Conductive polymer-based flexible sensors have been designed by incorporating conductive polymer sensor electrodes with soft substrates. Conductive polymer-based flexible array sensors are widely applied for many types of sensors and for detecting different analytes in various fields owing to their tunable robustness, low power consumption, real-time mapping ability, and good analytical performance. From this perspective, conductive polymer-based flexible sensors must adapt to certain requirements, such as simple and effective fabrication methods, fast responsivity (1–2 s), low cost, high sensitivity and present transparency, high stretchability, and good compatibility. Such devices can be designed as skin-like sensors using e-skin substrates that mimic human skin functions.

## Data Availability

The data presented in this study are available on request from the corresponding author.
